# Edema and Trismus Assessment After Applying Kinesiotape With the Web Strip Technique Compared With Cryotherapy: A Randomized Clinical Trial

**DOI:** 10.7759/cureus.66527

**Published:** 2024-08-09

**Authors:** Belal Alhourani, Mazen Zenati

**Affiliations:** 1 Oral and Maxillofacial Surgery, Damascus University, Damascus, SYR

**Keywords:** cryotherapy, web strip technique, kinesiotape, trismus, edema

## Abstract

Background and objectives: Extraction of impacted third molars is one of the most common oral surgeries performed in the dental clinic, which is often accompanied by many complications such as edema and trismus. Many methods have been used to alleviate these complications, such as drugs or physical therapy. Kinesiotape (KT) has recently spread as a popular physical method for eliminating complications after surgical extraction of lower third molars after its long-term use in sports medicine and injuries of the musculoskeletal system. The current study aimed to study the effect of using KT (Kinesio^®^ Holding Corporation, Albuquerque, NM, USA) on both edema and trismus after impacted third molar extraction.

Methodology: This study was designed as a randomized controlled clinical trial using the split-mouth technique and included 25 patients with radiographically symmetrical lower third molars from patients. All surgical extractions were performed by a single surgeon under sterile conditions according to the standard surgical protocol after that one group applied KT and the other group applied cryotherapy. Edema and trismus were measured in the first five days. Data was collected and analyzed by SPSS software (IBM Corp., Armonk, NY, USA).

Results: The mean change in the total linear facial measurements in the experimental group (Kinesio Group) was 12.32 mm three days after surgery, and then this value decreased to 6.80 mm, while the average increase in the control group (Cryo Group) was 17.00 mm after three days, then the value decreased to 9.68 mm five days after surgery. Regarding the changes in the amount of maximum mouth opening after surgery, the results were similar between the Kinesio Group and the Cryo Group, as there were no significant differences between the two groups (P<0.05).

Conclusion: This study concluded that KT was superior to cryotherapy when studying edema. The current study also concluded that the mouth opening was similar between the two study groups.

## Introduction

Extraction of impacted third molars is one of the most common oral surgeries performed in the dental clinic, which is often accompanied by many sequelae, such as edema and trismus, which vary in severity and impact on the patient’s quality of life [1]. Therefore, many pharmaceutical and non-pharmacological methods have been used to control and alleviate these complications [2] such as nonsteroidal anti-inflammatory drugs, corticosteroids [3], and compression bandages [4], but these methods have not shown great reliability in eliminating complications. The latest studies have indicated that the use of Kinesiotape (KT) can have a significant effect on reducing swelling and trismus after surgery [5].

KT was introduced by Dr. Kase in the 1970s and is a thin, flexible tape that extends up to 40-60% of its original length and is similar to the properties of skin in its thickness, weight, and ability to stretch [6].

KT has recently spread as a popular physical method for eliminating complications after surgical extraction of lower third molars after its long-term use in sports medicine and injuries of the musculoskeletal system with the aim of reducing pain and swelling after injuries and improving the functional performance of the joints [6].

KT is made of cotton and elastic fibers and may include a polyester composition in types applied to sensitive skin when higher tension on the tape is needed to obtain the desired result. The tapes are waterproof and air-permeable, do not thermoregulate the site of application, and can remain on the skin for four to five days [7]. The thickness, weight, and stretchability of the tapes are similar to the properties of the skin [8].

Cryotherapy is an easy-to-apply, inexpensive, and repeatable method that is useful in reducing surgical complications through its effect on blood circulation, which causes the narrowing of blood vessels and slow metabolism and cellular exchange. Applying ice cube packs had an important effect in reducing pain after surgical extraction of the lower third molars. However, its effectiveness in reducing complications remains controversial [9].

Therefore, most studies recommend applying cold compresses for 10-20 minutes alternately during the first 12-24 hours of surgery, with rest periods of the same or twice the duration [10].

There are several ways to evaluate edema following extraction of impacted third molars, including measuring the distance between reference points (three or four points), using a facial scanner, or MRI. The method of measuring distances between points is reliable and easy to apply [11].

However, the use of some of these methods may be associated with side effects on the patient, while the effectiveness of others remains questionable, so the current study aimed to study the effect of using KT on both edema and trismus after surgery.

## Materials and methods

Study design and sample

**Figure 1:**
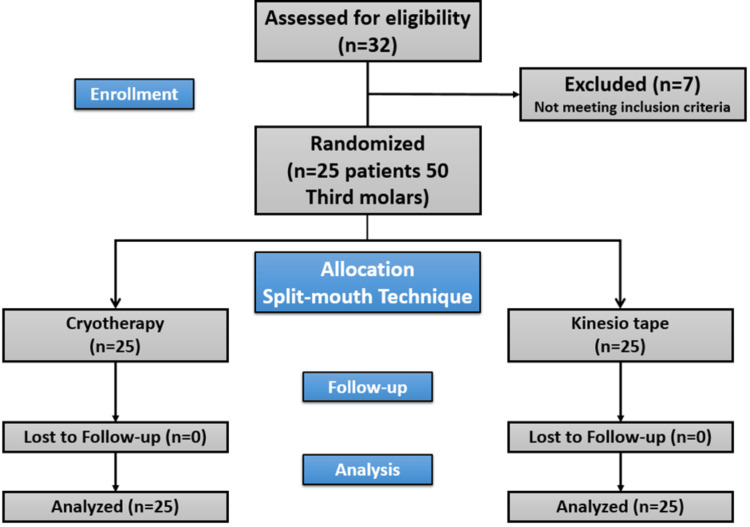
The Consolidated Standards of Reporting Trails (CONSORT) flow chart of patients in the study

This study was designed as a randomized controlled clinical trial using the split-mouth technique and included 25 patients with radiographically symmetrical lower third molars from patients attending the Department of Oral and Maxillofacial Surgery, Faculty of Dentistry, Damascus University. Damascus University Ethics Committee issued approval DN 290424-230. All patients provided written informed consent before being included in the study procedures, which was conducted by the Declaration of Helsinki. This study has been registered in the Clinical Studies Registration Database with the ID (ISRCTN97083158) and this article has been written with the Consolidated Standards of Reporting Trails (CONSORT) statement (Figure 1).

The sample was calculated based on the data of a pilot study containing five patients and used G*power software where the effect size was 1.1501 and the results were 18 samples in each group. We raised this to 25 to account for dropout of patients.

Eligibility criteria

Criteria for including patients in the research sample included bilateral symmetrical lower third molars according to the angulation of impaction and moderate difficulty in extraction according to the modified Pederson scale. The ages of the patients ranged between 18-40 years, the duration of the surgical procedure was between 20-40 minutes, and patient was healthy systemically.

The exclusion criteria were pericoronitis and symptoms of severe pain before surgery, patients who are allergic to the adhesive tape material and the medications used in the research, patients who have thick hair where the material was applied, smoking, uncontrolled systemic diseases, pregnant and lactating women, poor oral hygiene, patients with temporomandibular joint disorders.

Randomization

The study sample (50 mandible third molars in 25 patients) was distributed randomly by flipping a coin to choose the side in which KT will be applied and also to choose the side in which the extraction will begin, as the time interval in performing the surgical procedure varied between the two sides by four weeks.

Measured variables

Trismus

**Figure 2:**
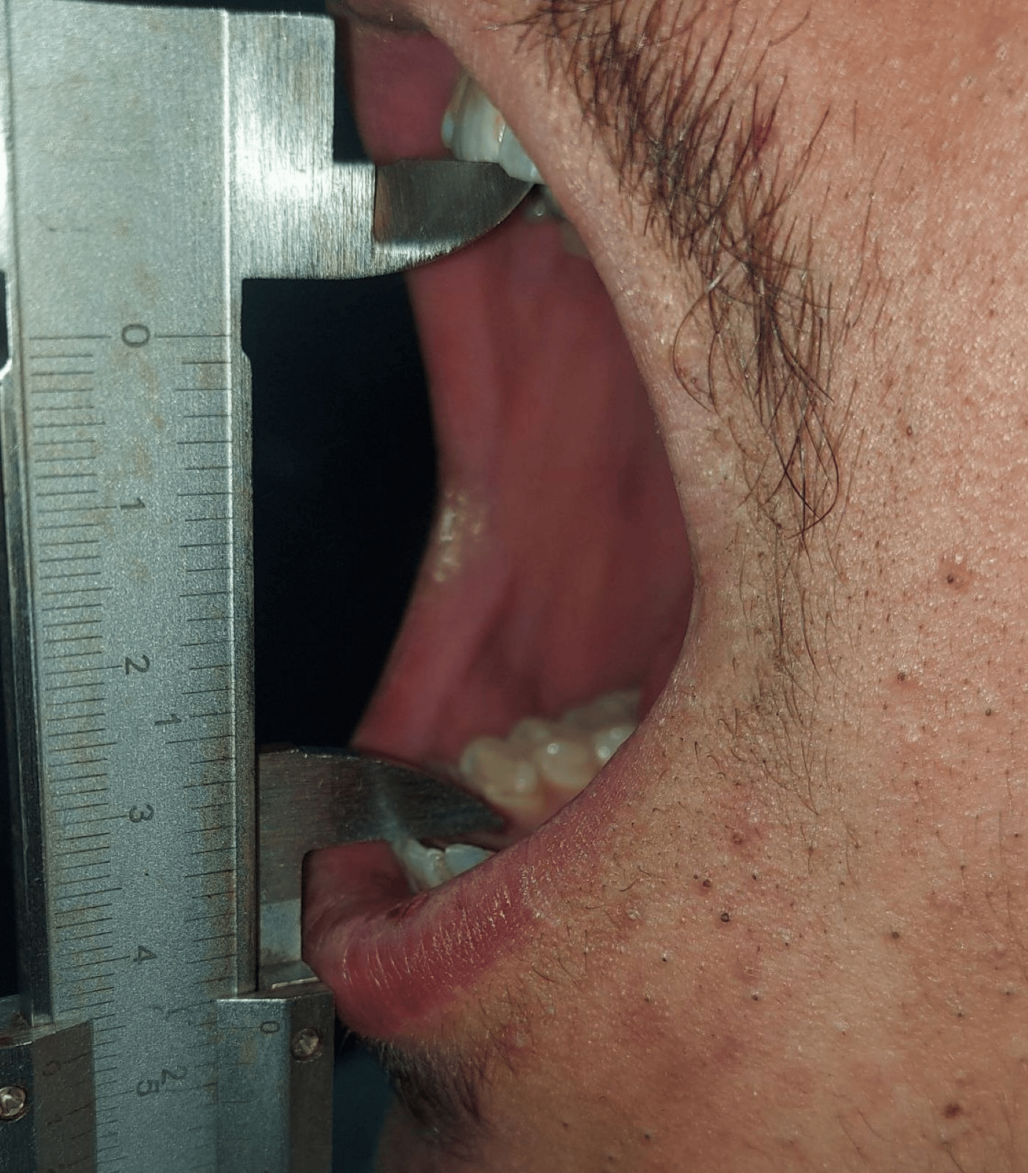
The measurement of the patient's maximum possible mouth opening

The distance between the incisal borders of the upper and lower centrals was measured with the patient's maximum possible mouth opening using a millimeter measuring tool (digital caliper) before surgery and on the third and fifth day after surgery to evaluate trismus according to the shape (Figure 2).

Edema

**Figure 3:**
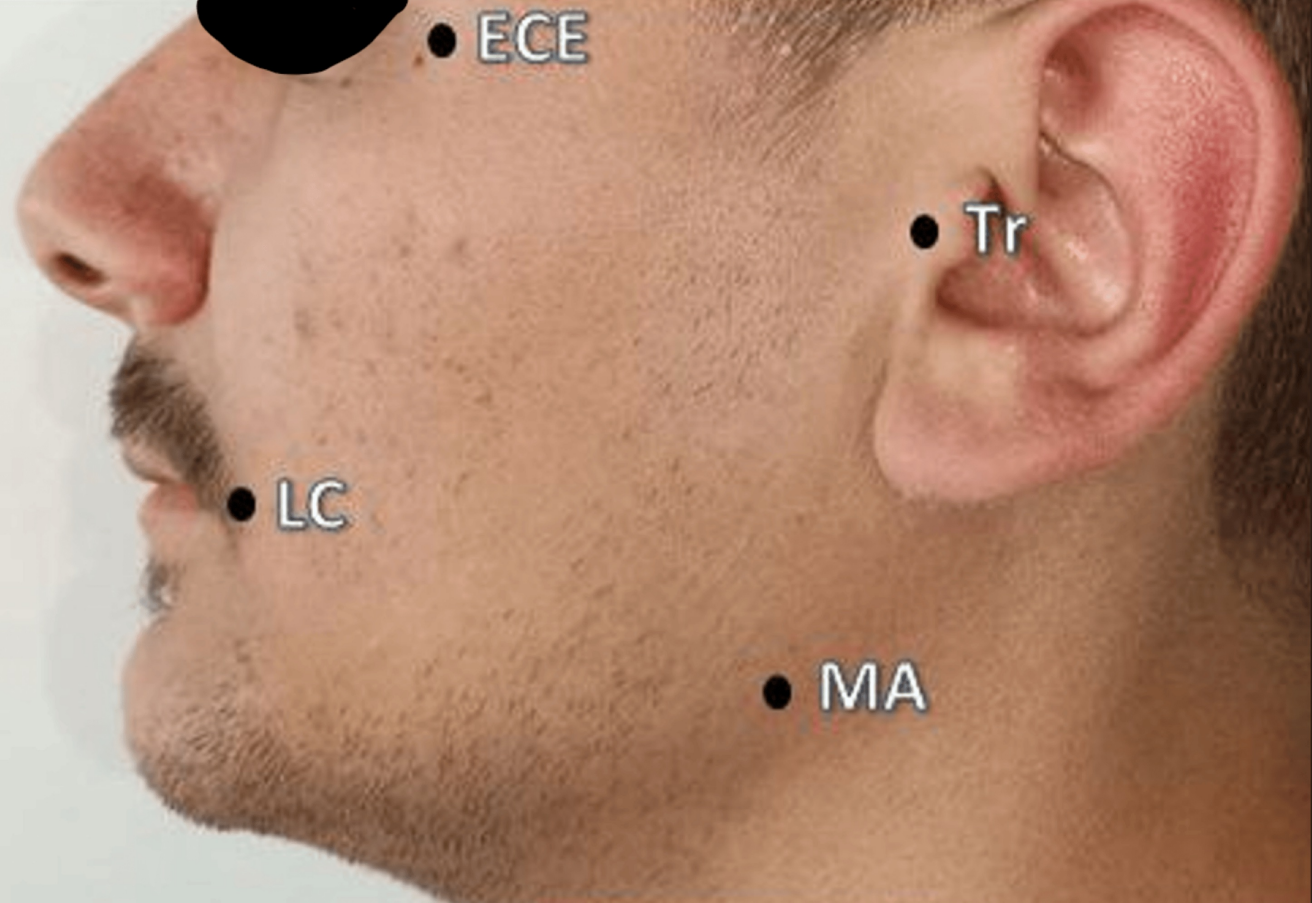
The measurement points of edema LC: oral commissure, MA: the angle of the mandibular, Tr: the tragus of the ear, ECE: the outer corner of the eye.

Facial swelling was evaluated preoperatively (T0) and on the third (T1) and fifth (T2) postoperative days using a millimeter measuring tool by measuring the lengths of the following four lines according to Figure 3 and calculating the resultant of their sum on each day of follow-up days. The first line is from the most lateral point of the oral commissure (LC) to the angle of the mandibular (MA), the second is from the most lateral point of the LC to the tragus of the ear (Tr), the third is from the MA to the outer corner of the eye (ECE), and the fourth is from the MA to the point of the Tr [12].

Surgical procedures

All surgical extractions were performed by a single surgeon (A.N.) under sterile conditions according to the standard surgical protocol as follows: a gingival triangular flap was elevated after nerve block anesthesia in the working area of ​​the inferior alveolar and lingual nerves and local anesthesia of the buccal nerve using lidocaine 2% with adrenaline 1/100,000. Then, the bone covering the tooth's crown was removed, and the tooth was separated in some cases when necessary, with continuous irrigation with saline. The tooth was wiggled and extracted using elevators, and then the flap was restored and sutured with 3/0 silk thread (VertMed GmbH, Syke, Germany).

Application of Kinesiotape

Kinesio Tex Gold Finger Print, 5 cm x 5 m (Kinesio Holding Corporation, Albuquerque, NM, USA) with a skin-like color (beige was cut to appropriate the recipient site as it is applied on the masseter area where most of the swelling occurs and where measurements are made between anatomical reference points using the “web strip” technique was applied immediately after completing the surgical procedure and suturing, after cleaning the skin and drying it.

The tension was adjusted by using the basic tension of the adhesive on the insulating paper of the original product so that we initially stick it to the skin and continue at the same pace to completely get rid of the insulating paper containing it.

**Figure 4:**
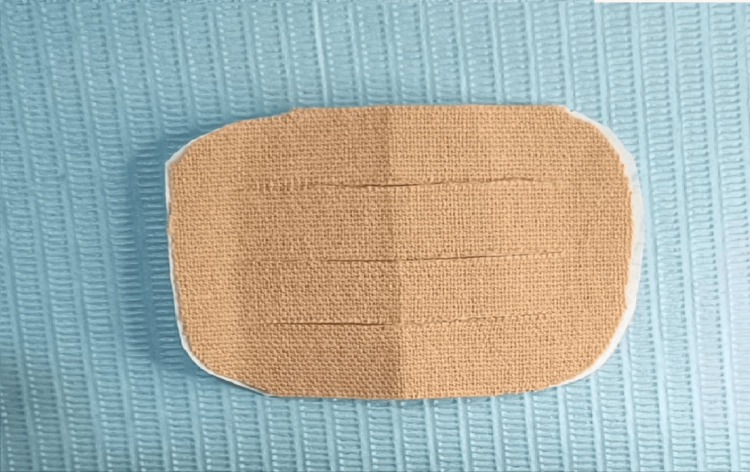
The web strip technique

The shape of the applied strip is modified according to the web strip technique (Figure 4) so that it has four elongations separated from each other in the center and connected at the two ends of the strip, in the same manner, following the Yurttutan and Sancak study [12].

After that, the tape adhered to the skin at the desired site using the basic tensile force applied to the tape with the insulating paper underneath (Paper Off), constituting 10-15% of the permissible tensile field of the tape without any excessive force.

The tape was placed on the skin for five days without any change. The patient was warned not to manipulate the tape or expose it to excessive water or moisture.

Application of cryotherapy

Cryotherapy was applied to the skin of the surgical site immediately after surgery during the first day for a period of 20 minutes application/20 minutes rest on the side to which KT was not applied using ice cubes in a cloth bag.

Post-surgical

All patients were prescribed amoxicillin (1g twice a day for seven days) and paracetamol (500 mg when necessary, provided that the daily dose does not exceed 3g) and to rinse with chlorhexidine twice daily (60 seconds) for seven days starting the day after surgery. The sutures were removed seven days after surgery.

Statistical analysis

Data were collected by one blinded examiner and entered into Excel (Microsoft, Redmond, WA, USA), after which the data distribution was studied and appropriate statistical tests were done using SPSS (IBM Corp., Armonk, NY, USA).

## Results

The number of patients participating in the research sample was 25. Patients had 50 third molars indicated for extraction, their ages ranged between 20 and 24 years (age mean = 21.52 ± 1.23). The patients were divided into 10 males (40%) and 15 females (60%) according to Table 1.

**Table 1:** Demographic data of the study sample

Group	Number of Extraction Sites	Male	Female	Age mean (years)
Cryo Group	25	10 (40%)	15 (60%)	21.52 ± 1.23
Kinesio Group	25			

No postoperative infectious complications occurred in the patients, and none of them had an allergic reaction during the application of KT.

Edema was studied by calculating the average of the sum of all four lines that we had previously determined in millimeters before surgery and on the third and fifth days after surgery.

Table 2 shows the descriptive data for the change in the sum of the four linear facial measurements at T1 and at T2 expressing facial edema in the Cryo Group and the Kinesio Group.

**Table 2:** Descriptive data for the changing in the sum of the four linear facial measurements T0: preoperative, T1: third day, T2: fifth day

Time of measurements (Days)	Cryo Group		Kinesio Group	
	Mean	Standard Deviation	Mean	Standard Deviation
T1-T0	17.00	±7.07	12.32	±6.76
T2-T1	9.68	±5.98	6.80	±5.81

The mean change in the total linear facial measurements in the Kinesio Group was 12.32 mm three days after surgery, and then this value decreased to 6.80 mm, while the average increase in the Cryo Group was 17.00 mm after three days, then the value decreased to 9.68 mm five days after surgery.

Table 3 shows the paired-sample T-test for the sum of the four linear facial measurements at T1 and at T2 expressing facial edema in the Cryo Group and the Kinesio Group.

**Table 3:** Paired-sample T-test for the sum of the four linear facial measurements *Significant difference P-value < 0.05 T1: third day, T2: fifth day

Time of measurements (Days)	Mean Difference	T value	P-value
T1	-4.68	-2.13	0.043*
T2	-2.96	-1.61	0.120

We noticed that swelling measurements were less in the study group in which KT was applied using the web strip technique than in the control group in which cryotherapy was applied on the third day after surgery, as the differences between the increased means in the total measurements were significant (P = 0.05 > 0.043), while there were no significant differences on the fifth day between the two groups (P<0.05).

Table 4 shows the descriptive data for change in mouth opening, where a negative value indicates a decrease in mouth opening and a positive value indicates an increase in mouth opening. These values ​​result from subtracting the amount of mouth opening before surgery from its amount at T1 and T2 in both groups.

**Table 4:** Descriptive data for change in mouth opening T0: preoperative, T1: third day, T2: fifth day

Time of measurements (Days)	Cryo Group		Kinesio Group	
	Mean	Standard Deviation	Mean	Standard Deviation
T1-T0	-17.40	±10.15	-17.30	±9.05
T2-T1	-9.24	±8.93	-9.68	±7.36

Table 5 shows the paired-sample T-test for the maximum mouth opening measurements at T1 and T2 expressing maximum mouth opening in the Cryo Group and the Kinesio Group.

**Table 5:** Paired-sample T-test for the maximum mouth-opening measurements *Significant difference P-value < 0.05 T1: third day, T2: fifth day

Time of measurements (Days)	Mean Difference	T value	P-value
T1	0.10	0.10	1.000
T2	-0.44	-0.39	0.695

Regarding the changes in the amount of maximum mouth opening after surgery, the results were similar between the Kinesio Group and the Cryo Group, as there were no significant differences between the two groups (P<0.05).

## Discussion

Edema and trismus are the most common complications that affect the patient’s quality of life after surgical extraction of the lower impacted third molars [13].

This study was designed according to the split-mouth technique, thus eliminating individual influences among patients. The effectiveness of applying KT was evaluated compared to applying cold packs, thus eliminating the placebo effect on the results.

Impacted lower third molars that were symmetrical in terms of angulation and moderate difficulty according to the modified Pederson scale were included in order to standardize the degree of difficulty of extraction and the surgical time between the two groups. Cases in which the difference in surgical duration between the two sides exceeded 10 minutes were also excluded [14].

In our study, we evaluated the effectiveness of applying KT using web strip technology compared to cryotherapy, as it is considered one of the simple and low-cost methods widely used in managing edema after surgery.

The measurement was done on the patient's face with a flexible, bendable ruler that does not use pressure on the skin. It is bendable to measure distance even in the presence of curvatures on the skin. It is also one of the methods approved in the study of Yurttutan and Sancak [12].

We did not perform surgical extraction of both sides in one session, because this may disturb the patient and hinder eating during the recovery period (four weeks).

Cryotherapy was applied immediately after surgery, and the patient continued to apply them at home during the first day, alternating between periods of application and periods of rest (20 minutes application/20 minutes rest), which is the method used in most studies [15].

Many methods have spread to measure facial changes resulting from swelling after surgery in the oral and maxillofacial area. Some of them lacked accuracy and others were costly. These methods include magnetic resonance imaging, 3D scanning, ultrasound, photographs, and linear measurements between reference anatomical points on the face [16].

In our study, linear measurements of the swelling variable and measurement of the amount of mouth opening were performed in the follow-up phase by an external observer to verify the accuracy and uniformity of variation in the measurements.

In Ristow et al. [17] and Jaron et al. study [18], KT was applied in the form of three separate strips between the clavicle bone and the line between the tragus of the ear and the oral commissure after surgical extraction of the impacted lower third molar. Gözlüklü et al. [14] presented a modified form of KT application to cover a larger area of the face and its effectiveness was compared with the application used in previous studies.

Yurttutan and Sancak [12] applied KT to a smaller area of the face in the chewing area, where most of the swelling appears after surgical extraction of the lower third molar, using the web strip technique to cut the tape. This reduces the aesthetic issues and the possibility of the patient's allergic reaction. All the techniques used to apply KT contributed to reducing swelling after surgical extraction.

In this study, we used the same method in applying KT as Yurttutan and Sancak [12], and no allergic reactions were observed in patients.

According to most previous studies, the duration of applying KT on the skin was five days. Therefore, the follow-up was made on the third and the fifth day.

In the past few years, interest in using KT in oral and maxillofacial surgery has increased. Tozzi et al. [8] reported the benefits of KT application in alleviating complications of orthognathic surgery. Coskun Benlidayi et al. [15] also reported its effectiveness in alleviating symptoms of temporomandibular joint (TMJ) disorders. The role of KT in reducing muscle pain caused by bruxism was reported in Keskinruzgaret et al. [16] and Ristow et al. [17] was among the first to apply KT after surgical extraction of lower third molars.

The mechanism of KT depends on the tensile force applied by the adhesive tape after it returns to its original length at the application site. This tension results in convolutions in the skin under the tape, increasing the interstitial distance between the skin and the connective tissues underneath, which reduces congestion and enhances blood circulation and lymphatic drainage in the area. This process has a role in draining fluids and moving them from areas of high pressure to areas of low pressure.

Swelling after surgical extraction of the lower third molars is at its maximum usually on the second to third day after surgery. It begins to disappear five to seven days after surgery.

In the study of Ristow et al. [17] Jaron et al. [18] and De Rocha et al. [19], swelling on the third day after surgical extraction of the lower third molar was lower in the KT group compared with the control group in which it was not applied, and this may be attributed to the effect of KT helps in lifting the skin and regulating lymphatic drainage.

Also, in the Yurttutan and Sancak study [12], designed with the split-mouth technique, in which KT was applied using the web strip technique after surgical extraction of the lower third molars, swelling was less in the KT group compared with the control group in which it was not applied.

In the Yuksel et al. study [20], it was noted that swelling was more in the KT group compared to the group in which cold packs were applied. However, this comparison was made after surgery knee joint replacement.

In our study, it was found that swelling on the third day after surgery was less in the KT group using the web strip technique than in the control group in which cold packs were applied.

In Ristow et al. [13], Tatli et al. [21], Chiang et al. [22], and Jaron et al. [18], the application of KT after surgical extraction of the lower third molars showed that the improvement of maximum mouth opening was faster compared to the control group in which KT was not applied. This may be due to the disappearance of swelling and pain faster in the KT group.

It was noted that trismus was reduced after surgical extraction of the lower third molar when applying KT using the web strip technique in Yurttutan [12].

In our study, there were no significant differences in trismus between the KT group and the control group, and this is consistent with the Tozzi et al. study [8]. This can be attributed to the lack of difference in pain between the two groups.

This study had some limitations, such as the inability to apply KT to males who did not want to cut their beard hair at the site of application.

## Conclusions

Edema and trismus are among the most important symptoms accompanying surgical extraction of third molars, and few of the techniques used have been effective in reducing these complications. The use of KT was one of the methods recently used to reduce these symptoms, and in this study it was compared with cryotherapy. This study concluded that KT was superior to cryotherapy when studying edema. The current study also concluded that the mouth opening was similar between the two study groups.
